# The role of regulation in the care of older people with depression living in long-term care: a systematic scoping review

**DOI:** 10.1186/s12877-020-01675-9

**Published:** 2020-08-05

**Authors:** Michelle Crick, Robin Devey-Burry, Jiale Hu, Douglas E. Angus, Chantal Backman

**Affiliations:** 1 University of Ottawa, Roger Guindon Hall, 451 Smyth Rd, Ottawa, Ontario K1H 8L1 Canada; 2 School of Nursing, Faculty of Health Sciences, University of Ottawa, Roger Guindon Hall, 451 Smyth Rd, Ottawa, Ontario K1H 8L1 Canada; 3 Department of Nurse Anaesthesia, Virginia Commonwealth University, Richmond, USA; 4 Telfer School of Management, University of Ottawa, 55 Laurier Ave E, Ottawa, ON K1N 6N5 Canada

**Keywords:** Depression, Older people, Long-term care, Regulation

## Abstract

**Background:**

This aim of this study was to explore the role of regulation on the quality of care of older people living with depression in LTC, which in this paper is a domestic environment providing 24-h care for people with complex health needs and increased vulnerability.

**Methods:**

We conducted a systematic scoping review. A peer reviewed search strategy was developed in consultation with a specialist librarian. Several databases were searched to identify relevant studies including: Embase (using the OVID platform); MEDLINE (using the OVID platform); Psych info (using the OVID platform); Ageline (using the EBSCO platform); and CINHAL (using the EBSCO platform). Articles were screened by three reviewers with conflicts resolved in consultation with authors. Data charting was completed by one reviewer, with a quality check performed by a second reviewer. Key themes were then derived from the included studies.

**Results:**

The search yielded 778 unique articles, of which 20 were included. Articles were grouped by themes: regulatory requirements, funding issues, and organizational issues.

**Conclusion:**

The highly regulated environment of LTC poses significant challenges which can influence the quality of care of residents with depression. Despite existing evidence around prevalence and improved treatment regimens, regulation appears to have failed to capture the best practice and contemporary knowledge available. This scoping review has identified a need for further empirical research to explore these issues.

## Background

The prevalence of depression in older people living in long-term care (LTC) is high [1–4]. Recognition of depression in older people living in LTC is problematic. First, it has been suggested that older people are less willing to report feeling depressed [5], and second, there is a risk that professionals believe it to be a normal part of the aging process [6, 7]. Depression has a significant impact on the well-being of older people living in LTC [8]. Depression in LTC is associated with loneliness [5, 9, 10], physical morbidity [11–13], failure to thrive [14, 15], and suicidality [16].

Regulation in LTC has been linked to quality [17–19], although there are other contradicting views which suggest it can distract LTC care workers from providing quality care to residents [18]. Lobel [20] suggests that control and command approaches to regulation, which focus on identifying what organizations fail to achieve, are ineffective, whereas Ferrino [17] reports responsive approaches, characterized by support and collaboration are more effective. Although regulation in LTC has increased [21], there remains significant issues in the provision of quality services in LTC settings. Concerns of poor quality in LTC are evidenced by reports and studies from the past 30 years related to the quality of care in the sector [22–24], which might suggest that attempts to improve quality by increased regulation is proving to be unsuccessful in many jurisdictions. This presents an urgent need for more reliable methods of addressing quality in LTC [25].

Many frameworks, models of care and evidence-based practices have been described in the literature to improve the quality of care for older people living with depression in LTC. Furthermore, multi-disciplinary approaches to care [26] which involve specialist teams have been suggested as good practice [27, 28].

Recognition of depression in LTC residents is a crucial aspect of quality [29], with assessment being the cornerstone of effective treatment [30]. Despite what is already known about the prevalence, recognition, and treatment of depression in LTC, implementing these best practices into regulation is challenging. In research exploring the alignment of quality improvement in care of depression in home care services, Bao et al. [30] found funding incentives for reimbursement were misaligned with best practices in the care of people with depression, specifically, a lack of explicit recognition of the amount of nursing time that is needed when supporting a person with depression. Nurses in this study were aware that they were unable to implement best practice but felt inhibited by policy that incentivized productivity. They also found the electronic health record system they were working with was a barrier to completing ongoing assessments, as the electronic system only allowed a brief window of time for uploading supplementary assessments. This resulted in nurses being unable to supplement any initial screening with a more detailed mental health assessment. Boyle et al. [31] concluded, that whilst it was used inconsistently in their study, participants using the Geriatric Depression Scale (GDS) [32] felt it would improve quality. Similarly, 90% of experts endorsed the use of the GDS when developing guidelines for treating depression in nursing homes [33].

There are risks from failing to recognize depression in LTC. Podgorski et al. [34] studied suicide risks in LTC facilities and found there were opportunities for suicide prevention through local risk management strategies as well using a population-based approach. The research team found that there were opportunities for LTC facilities to assess their own local populations and set local goals and priorities, according to their needs, thus being able to develop policies and goals which balance safety and risk, with the freedom and individual preferences of residents.

Although a deterrence approach to regulation dominates the LTC sector [17], other studies have described alternative approaches to achieving quality. These are characterised by participation, flexibility and responsiveness, collaboration and partnerships, dynamic learning, and self-enforced regulation [18, 20]. A study which explored the integration of best practice guidelines into system based protocols for quality monitoring in LTC, found a collaborative approach between educators and licensing agencies was a way to improve quality, whilst at the same time continuing to utilize the Minimum Data Set (MDS) [26]. However, some experts have found the MDS to be limiting in the field of depression [35], in part because its implementation in relation to psycho-social care had several barriers, including variation across jurisdictions and lack of federal guidance for its use [36].

An expert panel, which included leaders and stakeholders in the field of LTC, systematically reviewed the literature and made recommendations that families, residents, and ‘substitute decision makers’ (where capacity is an issue) should be actively involved in decision making in the LTC environment, as stakeholders [37]. LTC is a unique environment, given it is the residents’ home, and arguably regulations should reflect the preferences and individualities of the residents themselves [38, 39]. Lenhoff [35] led an expert review of quality in LTC and suggested that regulation should reflect the need to promote autonomy in residents.

In summary, the treatment of older people with depression living in LTC is hindered by several factors including, a lack of recognition of depression, an increased prevalence of depression and a reduced access to treatment for depression. The literature also shows that whilst LTC quality is driven by regulation across the sector, there remains concerns about quality. It appears there is strong evidence that collaboration and partnership is an effective way to deliver care in this setting. The concepts of regulation, long-term care and depression, have been extensively addressed in the literature in isolation, however, there is a paucity of literature examining the relationship between the role of regulation and the care of older people with depression who are living in LTC. The aim of this systematic scoping review was to describe and analyze published studies and relevant grey literature, to explore the role of regulation on the quality of care of older people with depression living in LTC. Whilst the authors acknowledge the aim of this scoping review may be somewhat ‘lofty’ and hard to address, beginning with a scoping review of the literature to examine what evidence is available, is a worthwhile starting point. A scoping review of the existing literature will allow us to be better able to identify gaps and inform future research in this area.

## Methods

This systematic scoping review is based on the Arskey & O’Malley’s Methodological Framework for Scoping Studies [40]. The complete detailed protocol is published elsewhere [41].

### Search strategy

A search strategy was developed in consultation with experts in both scoping reviews, and in the topic area, along with consultations with a specialist librarian who had knowledge of the health literature. Search terms were developed by capturing a broad notion of the different concepts of regulation, older people, depression, and LTC. LTC and nursing homes are terms often used interchangeably in the literature but for this scoping review only LTC facilities or nursing homes that provided “a domestic-styled environment that provides 24-hour functional support and care for persons who require assistance with ADLs and who often have complex health needs and increased vulnerability.” [42] were included. Depression was defined as a mood disorder, which causes severe symptoms that affect the individual emotionally and cognitively, which has an effect on daily activities, such as sleeping, eating, or working [43]. Regulation was characterised as standards set by governments or public agencies relating to activities valued by the community they serve [17].

To enhance the quality of the search strategy it was peer-reviewed by the specialist librarian using the ‘Peer Review of Electronic Search Strategies’ evidence-based guidelines [44], with subsequent changes incorporated into the search strategy terms. That said, quality assessments on the final articles, are not a required practice in scoping reviews [45], as this was not undertaken in this review. The aim of this scoping review was to explore the landscape of research available in this area to provide a basis for ongoing research, and as such, quality was not used as an exclusion criterion used in this scoping review.

Several databases were searched to identify relevant studies (Embase [using the OVID platform]; MEDLINE [using the OVID platform]; Psych info [using the OVID platform]; Ageline [using the EBSCO platform; and CINHAL [using the EBSCO platform]), using search terms which are listed in supplementary document 1. A search of the grey literature was also undertaken from various sources, using the concepts already described. The websites that were searched included Health Canada, Care Quality Commission, National Institute of Clinical Excellence, Manitoba Centre for Health Policy and Evaluation, health ministries, and the Canadian Institute for Health Information.

**Fig. 1:**
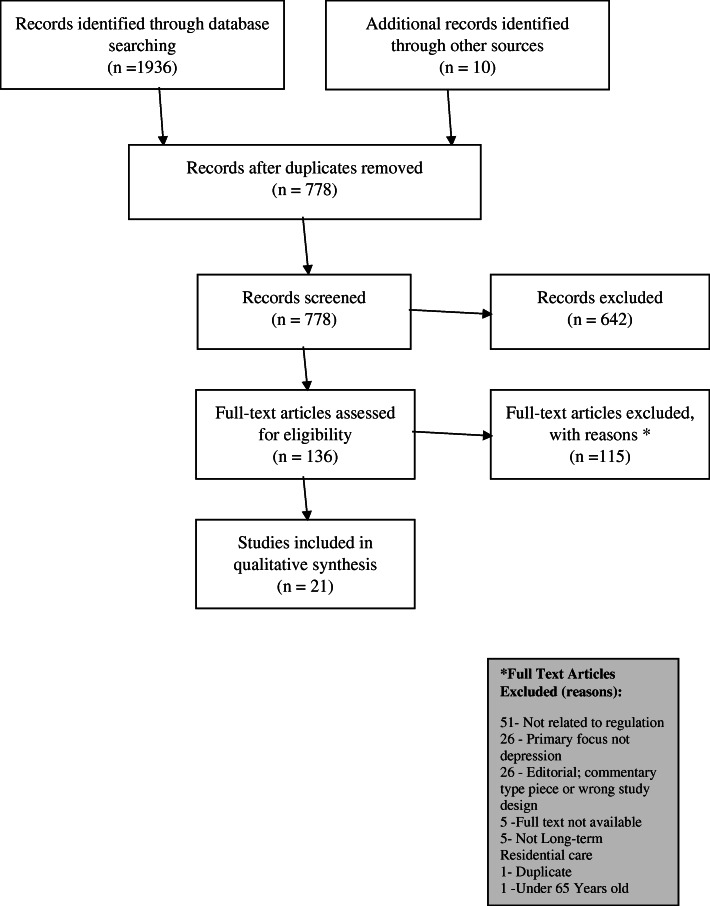
PRISMA flow chart – steps taken in the different phases of this scoping review

Using the broad concepts in the search strategy to identify items which were relevant, handsearching of relevant key journals, relevant reference lists and relevant grey literature identified from the systematic searches were also conducted. The Preferred Reporting System for Systematic Reviews and Meta-Analysis [46] was utilized to show the numbers of articles identified, screened, eligible and included in the study, see Fig. 1.

### Screening

A two-stage screening process was conducted using the pre-defined inclusion criteria. Any qualitative, quantitative, or mixed method studies focused on regulation, LTC, and depression were included. The inclusion criteria at the screening of title and abstract stage did not include age as a concept. This decision was reached in consultation with a specialist librarian, who agreed that adding age, as a concept, in the initial phase of the scoping review might limit results. Therefore, the concept of age was applied during the screening of full text articles only. Articles written in English and French were included in the study.

We excluded editorials, study protocols, and commentaries which only described policy or initiatives. Articles related to quality of life measurement, pain, palliative care, and cancer services were also excluded unless they were specifically linked to depression in LTC settings. There were no limitations placed on the dates of publications or the location of the studies in the search strategy. Covidence, an online systematic review software application, was used for this process.

A total of 1946 articles were retrieved from the search, with 778 remaining following removal of duplicates. In the first phase, three researchers (MC, RDB and JH) independently examined the 778 titles and abstracts produced from the systematic search. Six hundred forty-two articles were excluded in this initial screening based on information within the title and abstract of the article. Where this was unclear, or if the title and abstract did not provide enough information to decide, these articles were included in the full text arties which were assessed for eligibility in the second stage of the screening process.

Conflicts were noted in the outcome of this initial screening were resolved by consultation between the three reviewers (MC, RDB and JH) and other authors (CB & DA).

During the second stage of the process, the same group of researchers examined 134 full text articles, adding the concept of ‘older people’ (in this study described as 65 years or older). An article was included if it was related to regulation, older people, depression, and LTC.

### Data charting and analysis

**Fig. 2:**
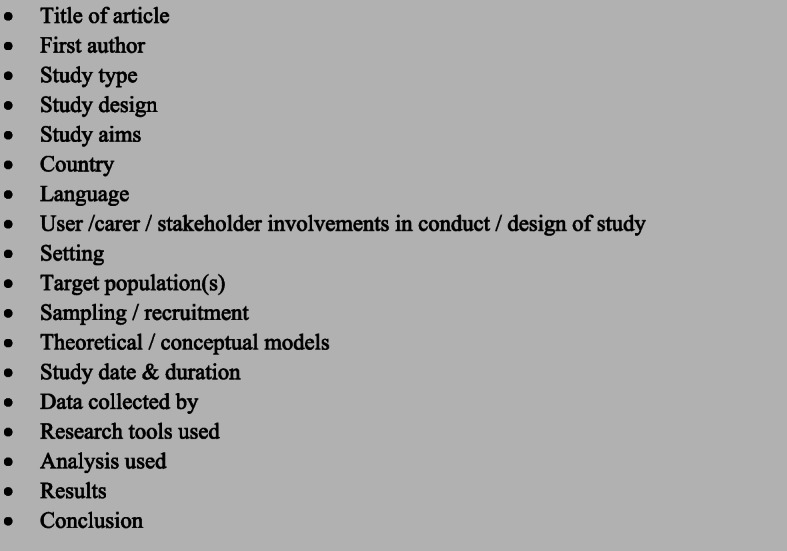
Charting Review Headings – heading for collating the content of full text articles

Data was charted using a tool based on Booth, Sutton & Papaionannou [47], which can be seen in Fig. 2.

Three of the articles were randomly selected and reviewed by a second reviewer (RDB) and checked for consistency and quality. Results were charted using an excel spreadsheet. A narrative synthesis was conducted which consisted of extracting key themes from the scoping review studies as well as exploring the relationships between the themes.

## Results

Of the final 20 articles included in this scoping review, there was 1 article each from France, Canada, the UK and New Zealand; 2 from the Netherlands; and 14 from the USA. This might be explained by the strong relationships in the USA between quality assessment and remuneration for health benefits, Medicare, and Medicaid which has required regulatory processes and an attempt at standardization with the RAI/MDS [36].

Chart 1 presents an overview of the full text articles by their primary topic area. Of the final 20 articles, many were focused on models of care and the assessment and treatment of depression. Evaluation studies and pharmacology also featured strongly. There were no restrictions placed on dates in the search strategy, and indeed, some articles are older. This explains how several articles were related to Omnibus Budget Reconciliation Act (OBRA) legislation, which was introduced in the USA in 1987, and which instituted screening for individuals placed in nursing facilities. OBRA (1987) legislation also influenced the development of the Resident Assessment Instrument (RAI) and MDS tool, which was linked to Medicare and Medicaid reimbursement [48], and which was also represented in several retrieved articles. Articles in this scoping review focused on the role of such legislation, may explain the presence of a selection of certain older articles retrieved in this scoping review.

Eleven of the studies had a quantitative design; 1 study was qualitative; 3 were reviews; and 5 had a mixed methodology approach.

Regulatory requirements; funding and organizational issues were identified as key themes in this scoping review.

### Theme #1: regulatory requirements

There are several different approaches to quality described in the literature, which suggest accreditation and regulation are linked to quality outcomes in care [17]. In many cases, the funding streams in LTC are linked to meeting predetermined performance measures, using tools such as the MDS or RAI to establish levels of need; measuring whether those needs are met; developing a case mix index; and apportioning funding according to the level of need of the LTC facility [49]. How well residents perceived needs are met was studied by Holtkamp et al. [50], who assert that there is a lack of research conducted which explores the difference between the residents’ perceptions of needs with those of the nurse. They investigated the effect of the RAI on the perceived level of need between residents and nurses, suggesting there were gaps. However, they did note that using the RAI was associated with improvements in meeting resident’s needs. Chodosh et al. [51] argue that the MDS improved opportunities for assessment and examination of nonpharmacological care but conclude in their study that a lack of technical knowledge and awareness of this tool could be a barrier to its effectiveness in LTC.

The extent to which systems which are governed by regulation in the LTC sector make a difference to the quality of care that is delivered has been studied. In the USA, OBRA (1987) was enacted with the goal of helping improve LTC residents’ quality of life by mandating standardized assessments; by prescribing a policy of psychotropic drugs; and by enacting care planning requirements [52]. This act led to the use of the Pre-Admission Screening and Annual Resident Review Program (PASARR), which was designed to improve access to appropriate mental health care for long-term care residents [53]. This scoping review found that the program had mixed reviews. Taylor et al. [52] explored prescribing patterns of psychotropic drug use before and after the implementation of the OBRA (1987) legislation in a not for profit LTC facility, using data retrieved from medical records. They found that before the enactment of the OBRA (1987) regulations, the use of anti-depressant medication was higher than after the introduction of OBRA (1987) legislation. However, they also noted that other concurrent legislation also influenced the success seen in psychotropic prescribing patterns, making it harder to credit success to OBRA (1987) which was introduced in the changing context of the healthcare landscape. For instance, in January 1994, Medicaid in Georgia, USA stopped reimbursing LTC facilities for prescribing anxiolytics (usually benzodiazepines) except where they were prescribed for the treatment of seizures. The Medicare and Medicaid system in the USA is an example of a federally regulated system which may present barriers for LTC residents in accessing specialised treatment. Linkins et al. [53] found reimbursement from Medicare and Medicaid was often too low to incentivise LTC facilities to organize appropriate mental health input from specialised teams, despite the provision of mental health care being mandated in OBRA (1987) [53, 54].

In Hanlon et al. [55] they noted that the Centre for Medicare and Medicaid Services included antidepressants in a list of potentially ‘unnecessary medications’, which may have had influenced the reduction in antidepressant prescriptions noted in their study of psychotropic drug use in the USA between 1996 and 2006. This study further demonstrates that concurrent regulations can influence decisions made at the local level. However, given what is known about prevalence of depression in LTC it was not clear why these drugs are included in this list, especially since best practice indicates Selective Serotonin Reuptake Inhibitors are a first line treatment of depression [56]. Other studies have seen less success in using the PASARR process to identify depression. Borson et al. [54] studied over 7000 residents in 54 facilities and found that whilst the PASARR process in LTC identified residents with schizophrenia, it was less successful in identifying those residents with depression. The seriousness of depression in LTC [56], is highlighted by the OBRA (1987) legislations’ requirement for potential residents to complete the Pre-Admission Screening Annual Resident Review (PASARR) prior to admission to LTC. PASARR was seen to be a feasible approach to determine whether nursing home applicants and residents required specialized services to meet their mental health needs [30]. It has been argued that these regulations have contributed to reducing inappropriate placements in USA nursing homes, by ensuring that nursing homes admitting residents with mental health issues are equipped with the necessary skills and the experience to provide care [30]. However, research indicates that OBRA (1987) has not enhanced the capacity of nursing homes to deliver appropriate mental health services, beyond standard case consultation and medication, suggesting some nursing homes cannot access appropriate mental health services for their residents [53]. Although the PASARR directives are mandated federally, Linkins et al. [53] noted significant variation in the implementation between different states and considerable latitude in defining ‘serious mental illness’, which is a key factor in the legislation.

Incorporating assessment in mental health care has been shown to have a positive impact on care [26, 30]. Molinari et al. [30] compared residents in not for profit facilities who had a mental health assessment conducted versus those who did not receive this assessment. They found that incorporating mental health assessment assisted with care planning, promoted non-pharmacological approaches to care and was quick and feasible. Murphy et al. [26] evaluated a program to manage depression in LTC and found that utilizing the MDS assessment in a more structured approach was helpful to staff when planning and implementing care, to improve residents’ depression. Although there is emphasis on the role of the MDS as a tool for assessment in many jurisdictions, there was no evidence seen in the literature retrieved in this scoping review regarding the importance of completing ongoing assessment of depression of residents in LTC, within regulatory frameworks. Datto et al. [57] found that MDS measures are sensitive to changes in depressive symptoms and as such would provide the means for ongoing monitoring; and evaluation of adherence to treatment in depression, in their study of pharmacological treatment of nursing home residents.

Various studies retrieved in this scoping review suggest there is variability in how such mandated tools are implemented across different areas. For instance, in a national study in the USA, Linkins et al. [53] noted that federal regulations allow for flexibility regarding how specialised mental health services are defined. Huang & Carpenter [58] researched use of the RAI tool in almost 500 residents in UK nursing homes. They found that government initiatives have not resulted in standardized assessment tools, resulting in an inconsistency of their application. A star quality rating system in the USA, which is based on health inspections, staffing, and quality measures, showed that a higher star rating scale was not associated with improving quality of life scores [59]. Similarly, in a UK study which explored the impact of using the Depression Rating Scale in LTC, found there was no association between a higher Care Quality Commission scores and lower rates of depression [58].

Holtkamp et al. [50] concluded from their study of 300 residents in Dutch nursing homes, that whilst using RAI leads to improvements in meeting of the residents’ perceived needs, they found that the implementation of RAI was varied, often due to staff absenteeism and turnover; and lack of computerization in the case of one area.

### Theme #2: funding issues

In this scoping review, almost one fifth of the articles were related to Medicare and Medicaid funding, the RAI, and legislation, which was not surprising since in many jurisdictions, performance, quality and funding are inter-related. In a study of over 88,000 residents in over 2000 facilities across 6 states in the USA, Lapane & Hughes [60] explored nursing home characteristics and their role in the management of depression. They found that when placement was funded from sources other than Medicaid or Medicare, a resident was more likely to be prescribed anti-depressant medication. To appreciate possible explanations for these findings, the characteristics of Medicare and Medicaid users’ needs further exploration. Kang-Yi et al. [61] studied the results of a Medicaid census and nursing home characteristics on quality of psychosocial care and found that higher Medicaid use in a LTC facility was associated with reduced recognition of psychosocial symptoms. Kang-Yi et al. [61] also noted that where staffing levels were improved, the prescribing of antidepressant medication was also more common. A study of almost 50 residents in Florida concluded that further understanding of funding was needed around mental health, with the authors asserting that Medicare and Medicaid are reluctant to reimburse for the costs of follow up mental health assessments [30]. Linkins et al. [53] concur, noting in their study of the impact of the OBRA (1987) legislation, the number of re-assessments conducted in LTC facilities were between 0 and 10%.

Wagenaar et al. [33] suggest formal guidelines and knowledge are available to guide practice, but that there are challenges to their implementation, including financial constraints, attitudes, and psychosocial barriers. They suggest feasibility is a significant issue when treating LTC residents who have depression. Some barriers, such as funding, exist because of complex reimbursement schemes [30], where follow up assessment for depression may not be funded by the state, or where the funding of anxiolytics has been curtailed except for specific medical conditions [52]. Linkins et al. [53] also suggest that regulation should be supported by the appropriate policies and financing to enhance the availability and integration of appropriate mental health services in nursing homes.

Most researchers argue that improved quality of care is linked to improvements in staffing levels [62], and the ratio of registered staff to residents [55, 60]. Recognition of depression has also been associated with having enough numbers of registered nurses in LTC homes that are able to identify the symptoms of depression in their residents. Lapane & Hughes [60] studied organizational characteristics of nursing homes and their influence on care of depression. They found that there was increased use of anti-depressants in facilities where there were more professional nurses. Trinkoff [63] studied data from over 15,000 nursing homes across 50 states, exploring the impact of Certified Nurse Aides (CNA) training on the quality of care provided, concluding that higher training hours were linked to better care outcomes; and that facilities offering CNA training above federally mandated hours resulted in fewer adverse events in the facility.

Linkins et al. [53] assessed the implementation of the PASARR program (was which part of the OBRA 1987 legislation), specifically exploring whether this had influenced the identification of Serious Mental Illness in nursing homes in the USA and found that funding was not always aligned to the new policy. As an example, federal statutes mandate that facilities are required to provide mental health services to residents with serious mental illness, however, in this study, 38 states reported that Medicaid only reimbursed LTC facilities for basic services for mental health. Linkins et al. [53] argue there is significant latitude at the state level, in the interpretation of this legislation, specifically with what is regarded as ‘basic services’. Holtkamp et al. [50] researched the implementation of the RAI in Dutch nursing homes, and found differences in how this tool was used, which would give cause for concern when the outcomes from using this tool are used to determine funding streams and case mix indices in LTC.

### Theme 3: organizational issues

Taylor et al. [52] suggests that nursing homes are subject to institutional and structural forces which can be challenging to staff, administrators, and policy makers, as they are faced with competing demands and priorities from funding constraints, inadequate staffing levels, care expectations and regulation. In a cross-sectional study of Medicare and Medicaid use in over 2000 nursing homes in 6 states in the USA, Lapane & Hughes [60] used MDS data from almost 88,000 residents to explore the relationship between nursing home characteristics and the management of depression. Findings from this study included how larger facilities were less likely to treat depression with anti-depressant medication, and how the structure and resources of the home influenced the choice of anti-depressant drug, with older tricyclic anti-depressant drugs used less in for profit facilities, in franchised facilities, and in homes with a higher percentage of Medicaid patients. The characteristics of LTC facilities also influenced the way in which care was organized. The articles retrieved in this scoping review showed that in LTC organizations with collaborative care models, there was a positive influence on the care of people with depression. Murphy et al. [26] describe, in positive terms, an intervention in which the Department of Health works alongside nursing homes to develop and implement best practices. Rolland et al. [64] examined the effects of an intervention which comprised professional support and education for nursing home staff linked to a range of quality indicators, including functional decline and emergency department transfers of residents. In their study they noted that improved communication and collaboration from the work between geriatricians and nursing home staff, improved problem solving when sharing psychiatric expertise, which ultimately had a positive influence on the care of depression in this population. However, in examining organizational issues in the relationship between regulation in LTC and depression, it is arguably harder to monitor quality when there is inconsistent application of tools and an inconsistent interpretation of the legislation [50, 53, 58]. Verkaik et al. [65] described the results of a small study of Dutch nursing homes, which explored the effect of introducing a nursing guideline for depression and dementia. They noted that its introduction had short term effects on how the CNA perceived their levels of autonomy, professionalism, workload and confidence levels. However, there were barrier to successful implementation to successfully implementing guidelines which included: time pressures, reorganization or other staff change, guidelines introduced in a ‘top-down approach’, the level of training of the CNA, lack of leadership, and unrealistic expectations that the guidelines would have an instant impact. Verkaik et al. [65] indicated that a multi-level, collaborative approach in translating guidelines to clinical practice was effective, and although leadership was identified by the team as enabling, the team working collaboratively was also perceived as a key to success. Trinkoff [63] found that nursing homes providing professional development time for CNAs which exceeded the minimum mandated requirement were linked to improved outcomes for residents. In terms of external support for LTC facilities, research has suggested that mental health services delivered to residents living LTC are inadequate [31, 50, 54]. Linkins et al. [53] found that over half the staff in their study had difficulty in accessing specialist mental health workers, who they found were unwilling to provide services to their residents in the nursing home, even when this had been identified as a need.

## Discussion

This systematic scoping review explored the role of regulation, and the care of older people with depression living in LTC. There is evidence that regulation in LTC does not necessarily have a positive role in the care of older people with depression. It could be argued that inspection processes result in LTC facilities being so concerned with what they need to demonstrate in relation to meeting standards, it results in a preoccupation with inspections leaving no time to think about quality [58, 59]. Such studies unavoidably lead to the question as to the value of such quality metrics, and whether residents and families can rely on them as a measure of what constitutes quality.

Many of the studies retrieved have presented issues relating to Medicare and Medicaid, which was not surprising since many articles in this scoping review were based in the USA. Although the articles explore the links between certain mandated requirements in different jurisdictions and depression in LTC, they did not explore the direct role of regulation on the care of people with depression living in LTC facilities.

Many of the articles in this scoping review have shown LTC to be a complex and highly regulated environment which has significant challenges due to funding constraints, and has many structures and processes in place which can influence the care of residents with depression. Despite existing evidence around prevalence and improved ways to manage depression in this population, generally regulation continues to adopt a deterrence-based approach [17], but which fails to incorporate into practice available contemporary knowledge [33, 65]. This scoping review also showed that there are many inconsistencies, in regulatory approaches in LTC [50, 53, 58, 59]. However, perhaps these inconsistencies in regulatory approaches in the sector enable organizations to meet regulatory standards in the context of funding being misaligned with what is required of the sector.

The widely used deterrence approach to regulation [17], may also fail to make the best use of learning opportunities, which arise from critical incidents in LTC, such as suicide, where in one study, organizational risk factors were rarely mention [66], which is a curious finding given that such a serious adverse event would almost certainly have organizational implications and learnings beyond the individual case. As with all research, there are limitations. One area which we might have explored further, to explore the influence of regulation, might have been to expand the search strategy to include other care environments.

## Conclusion

This systematic scoping review showed that in many cases decisions regarding regulation and legislative requirements in the LTC sector, have a direct influence on the structures and processes of care delivered to residents, but which staff, who are accountable for the provision of care, have little influence over. We have found a paucity of literature exploring the role of regulation on the care of older people living with depression in long term care. This suggests there is scope for further research which explores the role of the concepts of regulation on the care of older people with depression living in long-term care. The authors propose a study which can explore the relationships between these concepts in the long-term care setting and offer alternative models of regulation in the sector which could contribute to the policy direction of regulation.

## Supplementary information

**Additional file 1: Chart 1.** Overview of Articles – details of all full text articles which were reviewed in this study.

## Data Availability

Data sharing is not applicable to this article as no datasets were generated or analysed during the current study.

## Abbreviations

LTC: Long-Term Care
MDS: Minimum Data Set
OBRA 1987: Omnibus Budget Reconciliation Act (OBRA) of 1987
PASARR: Pre-Admission Screening and Annual Resident Review Program
