# Supplementary material for: The role of regulation in the care of older people with depression living in long-term care: a systematic scoping review
Source: BMC Geriatr. 2020 Aug 5;20:273. doi: 10.1186/s12877-020-01675-9 (PMC7409447; doi:10.1186/s12877-020-01675-9)
Supplement: Supplementary file 1 — Additional file 1: Chart 1. Overview of Articles – details of all full text articles which were reviewed in this study. [file 12877_2020_1675_MOESM1_ESM.docx]

| **Title of article** | **Psychiatric assessments of nursing home residents under OBRA-87: should PASARR be reformed? Pre-Admission Screening and Annual Review.** | **Recognition and management of depression in skilled-nursing and long-term care settings: evolving targets for quality improvement.** |
| --- | --- | --- |
| **First author** | Borson | Boyle |
| **Year** | 1997 | 2004 |
| **Country** | USA | USA |
| **Study type** | Quantitative | Quantitative |
| **Results** | Fewer than 10% of all nursing home residents were referred for psychiatric evaluation. | 26% of newly admitted nursing home residents had symptoms of depression that were apparent at admission, and an additional 12% were recognized early in their stay. Eighty-one percent of residents with depression were receiving treatment on admission to the facility, and 79% of those with depression recognized by Day 14 were treated by then. |
| **Statistical significance** | not applicable | not applicable |
| **Focus of article** | Implementation of PASARR | Assessment of Depression |
| **How was depression assessed?** | Had Depression Rating Scale (HDRS) | Geriatric Depression Scale (GDS) and clinical records |
| **Conclusions** | The role of cognitive deficits as a source of functional dependency and the need for nursing home care for patients with primary psychiatric illnesses plays a role in confounding clinical assessment of nursing home patients and contributes to the difficulty encountered in setting clear treatment goals and implementing effective management plans. | Depression is a significant issue for newly admitted nursing home residents; It should be a focus of ongoing quality improvement efforts in nursing homes; GDS is inconsistently used in nursing homes and supplementing the MDS with GDS would be helpful. |

| **Title of article** | **A Practice Improvement Education Program Using a Mentored Approach to Improve Nursing Facility Depression Care--Preliminary Data.** | **Pharmacologic treatment of depression in nursing home residents: a mental health services perspective.** |
| --- | --- | --- |
| **First author** | Chodosh | Datto |
| **Year** | 2015 | 2002 |
| **Country** | USA | USA |
| **Study type** | Quantitative | Review |
| **Results** | Depression scores did not change while medication use declined, from 37.2% of residents at baseline to 31.0% at 9 months (P < .001). |  |
| **Statistical significance** | medication use (P < .001) |  |
| **Focus of article** | Quality Improvement in Depression | Pharmacology |
| **How was depression assessed?** | The Minimum Data Set (MDS) Patient Health Questionnaire (PHQ-9) | Hamilton Rating Scale for Depression and the Cornell Scale |
| **Conclusions** | Assisting nursing homes with analysis of PHQ-9 data allowed strategic targeting of at-risk residents where interventions may make a significant difference; structured and monitored tools helped to ensure standardization across nursing homes | Issues that must be addressed to improve quality of care in nursing homes with depression should consider monitoring outcomes; modifying interventions; changing medication if first line treatments do not work well enough. Nursing home structures / care processes which have supported the increases in recognition of depression and initiation of treatment may not be enough to ensure on-going treatment and monitoring over the course of the illness. Treatment for depression should include non-pharmalogical strategies. Re-designed environments which improve autonomy and control for residents could improve depression. |

| **Title of article** | Improving the quality of age-related residential care through the regulatory process. | Antidepressant prescribing in US nursing homes between 1996 and 2006 and its relationship to staffing patterns and use of other psychotropic medications. |
| --- | --- | --- |
| **First author** | Ferrino | Hanlon |
| **Year** | 2013 | 2010 |
| **Country** | NZ | USA |
| **Study type** | Review | Quantitative |
| **Results** |  | Antidepressant prescribing significantly increased (P <.05) from 21.9% in 1996 to 47.5% in 2006. |
| **Statistical significance** |  | Antidepressant prescribing (P <.05) |
| **Focus of article** | Regulation | Pharmacology |
| **How was depression assessed?** | not applicable | not applicable |
| **Conclusions** | Recommendations in this report to support quality improvement make use of ideas or practices that have been implemented for many years in the US. The ideas suggested are universal and can be readily adopted in New Zealand with necessary modifications. Each also has ample evidence and research for New Zealand stakeholders to use when considering their implementation. The | Prescribing anti-depressant medication has increased in the past decade, these increases are associated with certain staffing characteristics and with co- prescribing of certain drugs |

| **Title of article** | **Effects of the implementation of the Resident Assessment Instrument on gaps between perceived needs and nursing care supply for nursing home residents in the Netherlands.** | **Identifying elderly depression using the Depression Rating Scale as part of comprehensive standardised care assessment in nursing homes.** |
| --- | --- | --- |
| **First author** | Holtkamp | Huang |
| **Year** | 2001 | 2011 |
| **Country** | Netherlands | UK |
| **Study type** | Mixed methods | Mixed methods |
| **Results** | Perceived gaps between needs and received care decreased more strongly in the experimental group (p<0.05) | Among the 499 residents assessed, 67.5% were not depressed (DRS<3) and 32.3% might suffer from depression (DRS>3). |
| **Statistical significance** | perceived gaps p<0.05 | not applicable |
| **Focus of article** | Assessment of Depression | Assessment of Depression |
| **How was depression assessed?** | Questionnaire was administered during a face-to- face interview with the residents exploring psycho-social needs, such as help needed in coping with feelings of sadness and depression, family problems and acceptance of physical deterioration | Depression Rating Scale (DRS) |
| **Conclusions** | Use of the Resident Assessment Instrument leads to better meeting resident needs | Depression Rating Scale has 3 merits when used observable rather than self-reporting; proxy scoring is allowed; is integrated into Resident Assessment Instrument |

| **Title of article** | **Interaction effect of Medicaid census and nursing home characteristics on quality of psychosocial care for residents.** |
| --- | --- |
| **First author** | Kang-Yi |
| **Year** | 2011 |
| **Country** | USA |
| **Study type** | quantitative |
| **Results** | The percentage of residents with depressive symptoms, low social engagement, and interpersonal relationship problems were 21.7%, 55.4%, and 10.1%, respectively. |
| **Statistical significance** | not applicable |
| **Focus of article** | Medicaid and Nursing Home Characterises |
| **How was depression assessed?** | Depression Rating Scale (DRS) |
| **Conclusions** | Nursing staff training in psychosocial assessment and well-being care, in particular is important. To obtain resources for training, nursing homes with high Medicaid census can collaborate with other nursing homes or social service agencies. Nursing homes with a high proportion of ethnic minority residents have lower level of detection rate for psychosocial well-being issues, and therefore culturally competent care should be a component of quality improvement plans. |

| **Title of article** | **The Association Between Quality of Care and Quality of Life in Long-Stay Nursing Home Residents with Preserved Cognition.** |
| --- | --- |
| **First author** | Kim |
| **Year** | 2014 |
| **Country** | USA |
| **Study type** | quantitative |
| **Results** | The average quality- of-life score was 71.4 (SD: 7.6; range: 45.1-93.0). Multilevel regression models revealed that quality of life was associated with physical impairment (parameter estimate=-0.728; P=.04) and **depression (parameter estimate=-3.015; P =.01)** but not Nursing Home Compare’s overall star rating (parameter estimate = 0.683; P =.12) and not pain (parameter estimate=-0.705; P =.47). |
| **Statistical significance** | p=0.01 |
| **Focus of article** | Quality of Life |
| **How was depression assessed?** | Participant Outcomes and Status Measured Nursing Facility survey |
| **Conclusions** | The 5-star quality rating system did not reflect the quality of life of long-stay nursing home residents with preserved cognition. Pain was not associated with quality of life, but physical impairment and depression were. |

| **Title of article** | **Which organizational characteristics are associated with increased management of depression using antidepressants in US nursing homes?** |
| --- | --- |
| **First author** | Lapane |
| **Year** | 2004 |
| **Country** | USA |
| **Study type** | Quantitative |
| **Results** | Increased treatment of depression with antidepressants was associated with facilities with a higher percentage of residents from payer sources other than Medicare/Medicaid (odds ratio [OR], 1.04; 95% confidence interval [CI], 1.02-1.06) and more professional nursing staff (OR, 1.15; 95% CI, 1.05-1.26). |
| **Statistical significance** | 95% CI |
| **Focus of article** | Organizational Characteristic in Nursing Homes and Anti-Depressant Use |
| **How was depression assessed?** | MDS |
| **Conclusions** | Few structural characteristics were associated with anti-depressant prescribing patterns; facility size was associated with anti-depressant and tricyclic anti-depressant use; Nursing homes with more beds were less likely to detect depression but if it was detected, more likely to treat depression with anti-depressant medication. Source of reimbursement is linked to anti-depressant use; findings highlight the need for interventions to consider resource and structural constraints in which nursing homes operate |

| **Title of article** | **Use of PASRR programs to assess serious mental illness and service access in nursing homes.** |
| --- | --- |
| **First author** | Linkins |
| **Year** | 2006 |
| **Country** | USA |
| **Study type** | Mixed methods |
| **Results** | Medical records showed that 50 percent of patients at the time of admission and 68 percent of patients at the time of the record review had a psychiatric diagnosis, typically a diagnosis of depressive disorder. Our findings showed that younger nursing facility residents were more likely to be given a diagnosis of any psychiatric illness (χ2=4.99, df=1, p<.05) |
| **Statistical significance** | p<0.05 |
| **Focus of article** | Effectiveness of PASARR |
| **How was depression assessed?** | PASRR level II screen, current prescription of psychotropic medications (for example, neuroleptics, antidepressants, anxiolytics, and mood stabilizers), or any psychiatric diagnosis |
| **Conclusions** | Compliance with the administration and documentation in PASARR was problematic. PASARR does show that nursing homes are not admitting excessively high numbers of people with serious mental illness. PASARR has not enhanced the nursing home’s ability to access specialist mental health services for residents |

| **Title of article** | **Influence of Mental Health Assessment on Prescription of Psychoactive Medication Among New Nursing Home Residents.** |
| --- | --- |
| **First author** | Molinari |
| **Year** | 2013 |
| **Country** | USA |
| **Study type** | quantitative |
| **Results** | Residents on psychoactive medication at admission I: 65 (15) C: 60 (15) (χ2 = 0.139, p = .709) Residents on insomnia medication at admission I: 22 (5) C: 8 (2) p=.175) Residents diagnosed with a mental illness at admission I: 57 (13) C: 44 (11) (χ2 = 0.751, p = .386) |
| **Statistical significance** | not statistically significant |
| **Focus of article** | Assessment and pharmacology |
| **How was depression assessed?** | Short Portable Mental Status Questionnaire and GDS |
| **Conclusions** | Systematic provision of mental health assessment and intervention may promote good quality mental health care by reducing psychoactive meds and increasing psychosocial interventions. This evidence-based approach could lead to reduced medical costs that offset the expense of any additional assessment |

| **Title of article** | **An initiative to improve depression recognition and management in long-stay nursing home residents.** |
| --- | --- |
| **First author** | Murphy |
| **Year** | 2005 |
| **Country** | USA (PA) |
| **Study type** | Qualitative |
| **Results** |  |
| **Statistical significance** |  |
| **Focus of article** | Recognition and Management of Depression |
| **How was depression assessed?** | Mood Resident Assessment Protocol and DRS |
| **Conclusions** | The identification of depression identification improved from 6.9% depression prevalence in July 2002 to 17% prevalence in March 2003; the leaders I the facility believe that depression is no longer expected, considered normal, or dismissed by staff. State certification and licensing agencies have traditionally viewed their role as limited to inspection and enforcement of regulatory requirements. Pennsylvania department of health initiative to develop and evaluate the use of best practice interventions and assist ‘average’ quality nursing homes to improve their care quality is unique among regulatory agencies. The Managing Depression Programme utilized the MDS assessment and care planning processes in a more structured, systematic way focusing on depression as a major quality problem. |

| **First author** | Rolland | Smith |
| --- | --- | --- |
| **Year** | 2016 | 2004 |
| **Country** | France | Canada |
| **Study type** | Quantitative | Review |
| **Results** | The intervention had a significant positive effect on the prevalence of assessment of pressure ulcer risk (p=0.006), **depression (p=0.02),** pain (p=0.03), and prevalence of ED transfers (p<.001). |  |
| **Statistical significance** | p=0.02 |  |
| **Focus of article** | Quality of Care | Quality of Life |
| **How was depression assessed?** | not applicable | not applicable |
| **Conclusions** | Audit feedback intervention combined with educational and professional support interventions can substantially improve healthcare quality in nursing homes. IQUARE shows a contribution from the collaborative work between hospital geriatricians & nursing home staff. Cost related to the intervention study would likely to be less than the cost of the unnecessary admission to the emergency room or hospital. The channels of communication post intervention showed an impact on pressure ulcer risk assessment, pain assessment. Communication improvements showed improved problem solving in palliative care & psychiatry. IQUARE has no effect on functional ADL decline. | this report focuses on five main areas for government action in the long-term care sector: improving quality of life; ensuring public accountability; developing clear enforceable standards with tougher inspection and enforcement; improving staffing and system administration; amending legislation and reviewing the funding formula |

| **Title of article** | **Psychotropic drug use in a nursing home: a 6-year retrospective.** | **CNA Training Requirements and Resident Care Outcomes in Nursing Homes.** |
| --- | --- | --- |
| **First author** | Taylor | Trinkoff |
| **Year** | 2003 | 2017 |
| **Country** | USA | USA |
| **Study type** | mixed methods | Quantitative |
| **Results** | After the Omnibus Budget Reconciliation Act of 1987 (OBRA) implementation, antipsychotic and antidepressant use was higher than at earlier data collection points, whereas anti-anxiety medication prescription was at its lowest. In addition, diagnoses of depression and cognitive impairment had increased dramatically by 1994. | A higher ratio of clinical to didactic hours was related to better resident outcomes. |
| **Statistical significance** | An ANOVA reveals that antidepressant prescription rates were at their highest at Time 3 (p = .047), whereas anti-anxiety prescription rates (p = .013) were at their lowest. | p<0.05 |
| **Focus of article** | Pharmacology | Certified Nurse Aide Training |
| **How was depression assessed?** | MDS | MDS |
| **Conclusions** | Initial analysis shows that OBRA 1987 is being translated into practice, but nursing homes are subject to structural forces which defy attempts to provide care (such as staffing; funding; regulatory requirements; unrealistic expectations from families); and advocates of changes do not notice the unseen ramifications | additional training providing clinical experiences may aid in identifying residents at risk |

| **Title of article** | **The introduction of a nursing guideline on depression at psychogeriatric nursing home wards: Effects on Certified Nurse Assistants.** |
| --- | --- |
| **First author** | Verkaik |
| **Year** | 2011 |
| **Country** | Netherlands |
| **Study type** | Quantitative |
| **Results** | The guideline introduction had a small, significant, positive effect on generally perceived professional autonomy in the Certified Nurse Assistants of the experimental wards (p<0.05). |
| **Statistical significance** | p<0.05 |
| **Focus of article** | Guideline introduction |
| **How was depression assessed?** | Provisional Diagnostic Criteria for Depression of Alzheimer disease and GDS |
| **Conclusions** | The nursing guideline had a positive effect on professional autonomy; |

| **Title of article** | **Treating depression in nursing homes: practice guidelines in the real world.** |
| --- | --- |
| **First author** | Wagenaar |
| **Year** | 2003 |
| **Country** | USA |
| **Study type** | Mixed method |
| **Results** | For both major and minor depression, over 70% of the group endorsed suicidal ideation and/or attempts, anxiety symptoms, dementia symptoms, psychotic symptoms, and medical comorbidity as important information to obtain during an evaluation. |
| **Statistical significance** | not applicable |
| **Focus of article** | Treatment of Depression |
| **How was depression assessed?** | MDS, GDS, the Center for Epidemiologic Studies Depression Scale and the Cornell Scale for Depression in Dementia |
| **Conclusions** | There are several discrepancies between importance and feasibility of combined modes of therapy, ECT, and psychotherapy. Experts endorsed the importance of these treatment modalities but did not see them as being feasible within a nursing home setting. Barriers include clinical access and financial, attitudinal, and patient-selection criteria. The MDS mood subscale poorly correlates with other well-proven assessment measures. However, the GDS was viewed as important but not feasible. The formal guidelines and recommendations issued by public and private organizations may not fit real-world patients, leaving clinicians confused about how to apply such guidelines effectively. |
